# Aflatoxin exposure and mortality in acutely ill children: results from the CHAIN network cohort

**DOI:** 10.1136/bmjgh-2024-017375

**Published:** 2025-07-17

**Authors:** Lei Xia, Hang Wu, Ali Faisal Saleem, Ezekiel Mupere, Christina Lancioni, Hama DIALLO, Isabel Potani, Syed Asad Ali, Wieger Voskuijl, Mohammod Jobayer Chisti, Abu Sadat Mohammad Sayeem bin Shahid, Molline Timbwa, Shalton Mwaringa, Caroline Tigoi, Moses Ngari, Benson Singa, Kirkby D Tickell, James Njunge, Robert Bandsma, Tahmeed Ahmed, James Berkley, Judd L Walson, Yunyun Gong, Michael N Routledge

**Affiliations:** 1 School of Food Science & Nutrition, University of Leeds Faculty of Engineering and Physical Sciences, Leeds, West Yorkshire, UK; 2 School of Medicine, University of Leeds Faculty of Medicine and Health, Leeds, West Yorkshire, UK; 3 Pediatrics & Child health, The Aga Khan University, Karachi, Sindh, Pakistan; 4 Makerere University, Kampala, Kampala, Uganda; 5 Uganda Case Western Reserve University Research Collaboration, Kampala, Uganda; 6 Pediatrics, Oregon Health & Science University, Portland, Oregon, USA; 7 Public Health, Universite Joseph Ki-Zerbo, Ouagadougou, Centre, Burkina Faso; 8 Paediatrics and Child Health, Kamuzu University of Health Sciences, Blantyre, Southern Region, Malawi; 9 The Hospital for Sick Children, Toronto, Ontario, Canada; 10 Pediatrics, Amsterdam University Medical Centres, Amsterdam, Netherlands; 11 Nutrition and Clinical Services Division, International Centre for Diarrhoeal Disease Research Bangladesh, Dhaka, Dhaka District, Bangladesh; 12 Clinical Research Department, KEMRI-Wellcome Trust Research Programme, Kilifi, Kenya; 13 Centre for Global Health & Tropical Medicine, Oxford University, Oxford, UK; 14 KEMRI, Nairobi, Nairobi County, Kenya; 15 Department of Global Health, University of Washington, Seattle, Washington, USA; 16 KEMRI-Wellcome Trust Research Programme, Kilifi, Kenya; 17 Cardiology, ICDDRB, Dhaka, Dhaka District, Bangladesh; 18 Centre for Tropcial Medicine & Global Health, Oxford University, Oxford, UK; 19 Global Health, Medicine & Pediatrics, University of Washington School of Public Health, Seattle, Washington, USA; 20 International Health, Pediatrics & Medicine, Johns Hopkins University, Baltimore, Maryland, USA; 21 School of Food and Biological Engineering, Jiangsu University, Zhenjiang, Jiangsu, China; 22 Leicester Medical School, University of Leicester, Leicester, UK

**Keywords:** Global Health

## Abstract

**Background:**

Chronic exposure to aflatoxins is associated with liver cancer, impaired child growth, and compromised immune function. The Childhood Acute Illness and Nutrition (CHAIN) Network cohort was established to identify risk factors for mortality in acutely ill children admitted to nine hospitals in four African and two South Asian countries. We examined the role of aflatoxin exposure in inpatient and post-discharge mortality.

**Methods:**

In a nested case-cohort from the CHAIN cohort, we compared aflatoxin exposure at admission and discharge with death or survival in hospital (n=755) or up to 180-days post-discharge (n=585) and with community participants (CP, n=222). Children were stratified into non-wasting, medium-wasting and severe-wasting groups based on mid-upper arm circumference. Serum samples were analysed for an aflatoxin exposure biomarker, the aflatoxin-albumin adduct (AF-alb) using ELISA.

**Findings:**

Overall, 56% of hospitalised participants tested positive for AF-alb at admission. The AF-alb level was higher in deceased (geometric mean and 95% CI (GM and 95% CI) 5.9 (4.9 to 7.1)) than in survivors (4.2 (3.8 to 4.7)) and CP (3.7 (3.1 to 4.3)) pg/mg alb. AF-alb concentration was higher at admission (4.7, (4.2 to 5.1)) than at discharge (3.7, (3.3 to 4.1)) and in the CP group (3.7, (3.1 to 4.3)) pg/mg alb (p<0.01) and in African vs Asian children (7.4 (6.5 to 8.5) vs 1.9 (1.8 to 2.1)) (p<0.001). Adjusted logistical regression showed no significant association between AF-alb levels and mortality, but after separating the nutrition strata, AF-alb was significantly associated with mortality (highest vs lowest quartile group OR=4.84, p=0.014) in non-wasted children.

**Interpretation:**

Moderate to severe malnutrition is a more important risk factor for mortality than aflatoxin in acutely ill children, but aflatoxin exposure may contribute to mortality in non-wasted children. Controlling aflatoxin exposure should be integrated into clinical and public health interventions to reduce mortality in areas with high levels of exposure.

**Funding:**

Bill and Melinda Gates Foundation (OPP1131320 & INV-003225).

**SUMMARY BOX**

- It is known that acute high dietary exposure to aflatoxin causes severe disease and may be fatal.
- It has not been shown whether lower levels of aflatoxin may contribute to mortality in severely ill malnourished children.
- This study has found that in severely ill children admitted to hospital, higher levels of aflatoxin exposure are associated with mortality, although severe malnutrition makes a bigger contribution.
- This highlights the need to reduce aflatoxin exposure in children and consider exposure as a risk factor for mortality in severely ill children in sub-Saharan Africa.

## Introduction

 High mortality rates have been reported for children under 5 years old from low- and middle-income countries (LMIC), particularly in sub-Saharan Africa and South Asia.^1^ Underlying comorbidities such as malnutrition, limited healthcare access and resources, poor quality food and poverty can all contribute to high mortality rates.^2^ Clinical severity of the illness and poor access to hospital treatment are key to high mortality. Reports showed that around half of all mortality happens after discharge for those children admitted to hospital for acute illness.^3 4^ The Childhood Acute Illness and Nutrition (CHAIN) Network is designed to study the determinants and mechanisms associated with death among acutely ill children in sub-Saharan Africa and South Asia.^5^

Aflatoxins are mainly produced by *Aspergillus flavus and A. parasiticus*, which favour hot and humid climate conditions.^6^ Aflatoxins can contaminate foods in the field before harvest and also due to poor post-harvest related storage conditions.^7^ Aflatoxin B_1_ (AFB_1_) and aflatoxin natural mixture (AFB_1_, AFB_2_, AFG_1_, AFG_2_) are Group 1 human carcinogens^8^ associated with increased risk of hepatocellular carcinoma.^9 10^ Acute high AFB_1_ exposure can cause liver damage and a high rate of fatalities.^11^ AFB_1_ has also been found to be associated with child growth impairment, suppression of immune function and hepatomegaly.^12^,^15^

Outbreaks of acute aflatoxicosis occur regularly in Africa, including outbreaks in Kenya in 1980, 2004 and 2005, and in Tanzania in 2016, which together caused hundreds of deaths. In addition, high levels of aflatoxin contamination in food are still reported across sub-Saharan and south Asian countries.^16^,^19^ As the high mortality rates for acutely ill children coincide with the high occurrence of aflatoxin exposure in sub-Saharan African and south Asian countries, it is important to evaluate the possible contribution of aflatoxin exposure towards the mortality rates for acutely ill children. Taking advantage of an established CHAIN cohort nested case control study (CNCC), this study aims to investigate whether aflatoxin exposure is associated with mortality among acutely ill children from sub-Saharan African and south Asian countries.

## Methodology

### Study participants: CHAIN and CNCC study set

The details of the CHAIN cohort and CNCC designs are published elsewhere, including participating sites, patient and public involvement, eligibility, screening, enrolment and follow-up procedures, specimen collection and selection of cases, and controls.^2 5^

**Figure 1:**
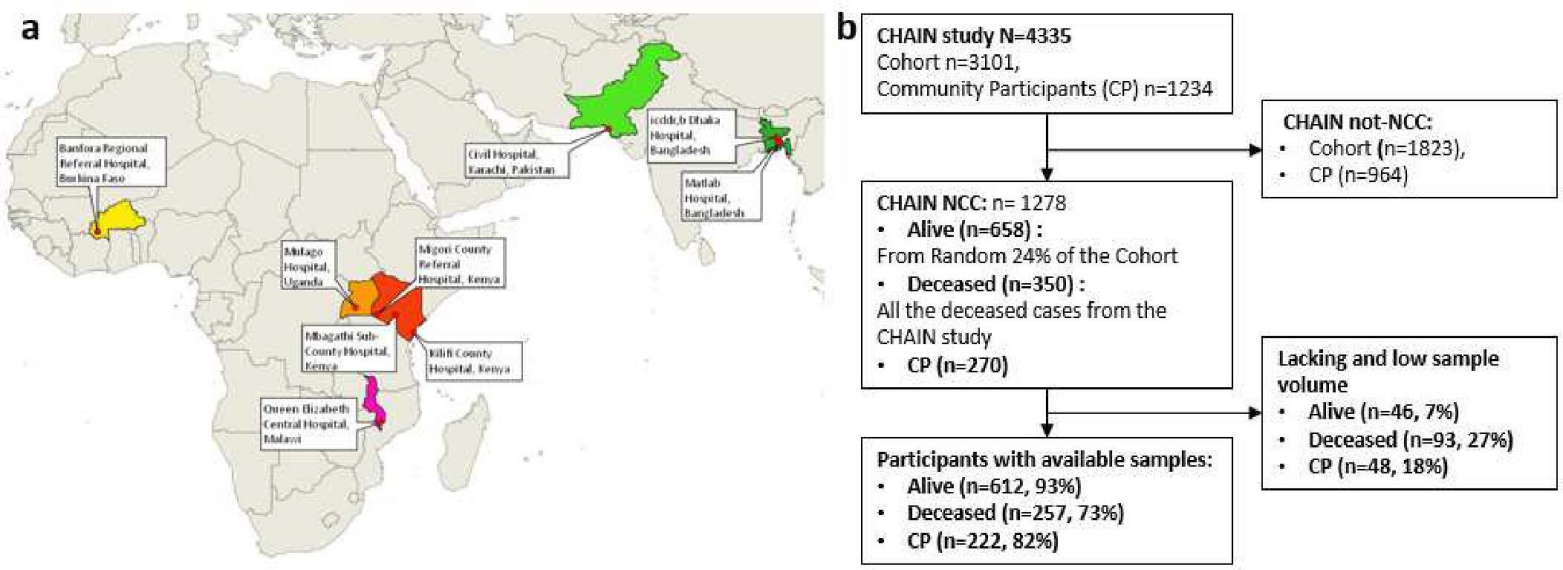
The study design. a. Geographic location of study sites which includes: Bangladesh (icddr,b Dhaka Hospital and Matlab Hospital), Burkina Faso (Banfora Regional Referral Hospital), Kenya (Kilifi County Hospital, Mbagathi Sub-County Hospital and Migori County Referral Hospital), Malawi (Queen Elizabeth Central Hospital), Pakistan (Civil Hospital, Karachi), and Uganda (Mulago Hospital); b. Schematic diagram for the study design and analyzed samples.

Briefly, 3101 acutely ill children were recruited from nine sites in six countries in sub-Saharan Africa and South Asia as shown in Figure 1.^4^ Children were enrolled at admission to hospital and followed up for 180 days after discharge from hospital (CHAIN BMJ protocol).^7^ The mid-upper arm circumference (MUAC) was used to categorise enrolled children into three strata that included not wasted (NW, MUAC ≥12·5 cm for age ≥6 months or MUAC ≥12 cm for age <6 months), moderately wasted (MW, MUAC 11·5 to <12·5 cm for age ≥6 months or MUAC 11 to <12 cm for age <6 months) and severely wasted or kwashiorkor (oedematous malnutrition) (SWK, MUAC <11·5 cm for age ≥6 months or MUAC <11 cm for age <6 months or bilateral pedal oedema unexplained by other medical causes). In addition, community participants (CPs) in the same communities as the hospitalised cohort, but who were not acutely ill, were recruited over the same time period (across one to 2 years) to establish community norms for demographic and biological factors.

The CNCC was set up to generate mechanistic insights into biological pathways leading to mortality, including those driven by infectious, nutritional and social factors, as has been previously described.^2^ Briefly, a random 24% sub-cohort of children was selected from the CHAIN cohort which included 720 survivors (CNCC controls) and 109 deaths. Thereafter, all remaining deaths (n=241 cases) that were not selected in the random 24% sub-cohort were added, giving a total of 350 deaths (CNCC cases). Additionally, 30 randomly selected CPs from each site (total n=270) were chosen. However, laboratory analysis of serum aflatoxin-albumin was only possible for a subset of the selected participants, which included admission (n=75), discharge (n=585) and community (n=222), due to participants lacking samples and those with insufficient (< 250 µL) sample volume (figure 1). These included admission samples for 233 deaths and 522 survivors, and discharge samples for 98 deaths and 487 survivors, and 222 CP samples.

### Patient and public involvement

This study concerned children under 2 years old and, therefore, was informed by observed patient outcomes rather than patient opinions. A community advisory board at each site reviewed the protocol and gave feedback to the investigators. The community advisor boards gave advice on appropriate community sensitisation and recruitment materials. Results will be disseminated at each site locally.

### Aflatoxin B_1_-albumin adduct (AF-alb) analysis

Serum samples stored at the KEMRI-Wellcome Trust Research Programme biorepository in Kilifi, Kenya, were shipped to the University of Leeds, UK, and subjected to AF-alb analysis.

The analysis of AF-alb followed the method described by Chapot and Wild^20^ with minor modifications.^21^ In brief, 2 mg of albumin, extracted from 250 µl serum sample, was hydrolysed overnight at 37°C using 0·67 mg of pronase (Boehringer, UK) and purified with a Sep-pak C-18 cartridge (Waters, UK). AF-alb was quantified using an indirect competitive ELISA method. The ELISA plate was coated with AFB_1_-ovalbumin adduct at 37°C overnight. Then the coated plates were washed using phosphate buffer saline-Tween 20 followed by blocking with skimmed milk. The standards, samples or controls pre-mixed with the rabbit anti aflatoxin-Cl_2_-BSA polyclonal antibody (a gift from Christopher Wild, IARC) were applied onto the ELISA plate and incubated for 90 min. After washing, the bound primary antibody was detected by incubation with a horseradish peroxidase enzyme labelled goat anti-rabbit secondary antibody. The limit of detection (LOD) of the ELISA assay is 3 pg AF-alb equivalents per mg albumin.

Quality controls: For each batch of sample, three positive and one negative quality controls were included. All samples were measured in duplicate each day and repeated at least two times on separate days. Results were accepted when the coefficient of variation was below 15%.

### Statistical analysis

As AF-alb data are not normally distributed, natural log transformation was performed to normalise the data for analyses. Positive rates (>LOD), geometric means (GMs) and 95% CI and ranges were tabulated. For concentrations below the LOD, a value of 1/2 LOD (1·5 pg/mg) was assigned for statistical calculation.^22^ Independent t-test or analysis of variance was used to compare the AF-alb concentrations in different groups. Paired t-test was used to investigate the difference in AF-alb concentration at admission and discharge.

In this study, demographic data including age, sex and study site, socio-economic status, health outcome (dead/alive and malnutrition status) and dietary consumption data with food frequency questionnaires (FFQ) were included in the analysis. The diet diversity score (DDS) was calculated based on a basic FFQ, whereby each category of food was recorded as one if consumed and 0 if not.

The DDS was calculated using the sum of food types that the child would eat on a typical day. Food type is defined based on WHO category, which includes milk and milk products, cereals and cereal products, fish and sea foods, roots and tubers, vegetables, fruits, meats and poultry, eggs, pulses/legumes/nuts and seeds, fats and oils, sugars/honey/commercial juices.^23^ Consumption of breast milk was not included in the calculation of DDS. In total, there are 12 categories, excluding breast milk; hence, the DDS ranges 0–12.

The household exposure domain (HED), an indicator of household wealth, was calculated using principal component analysis of household asset index.^6^

For the analyses of aflatoxin exposure determinants, food intake frequency, gender, HED, geographical site and weaning status were included in the logistic regression model. Age was not included as it is strongly associated with weaning stages. Logistic regression was used to investigate the association between AF-alb concentrations and death outcomes, adjusted by age, gender, geographical site, HED and nutritional strata in the full model. Subsequently, logistical regression was conducted in the NW, MW and SWK groups separately to test the independent relationship between aflatoxin exposure and mortality. The results used in the logistic regression analysis for the association between AF-alb and mortality were weighted according to the ‘CHAIN NCC weighting strategy’ (online supplemental material) to adjust for participant selection into the CHAIN cohort and then selection into the CNCC.

All analyses were carried out using IBM SPSS Statistics version 26, and a p value of less than 0.05 was used to assign for statistical significance.

## Results

### Demographic characteristics of the participants

Demographic data of the children in the study (at admission) are summarised in table 1. In total, 1091 participants (aged between 2 and 24 months, 57% male) from the CNCC study had sufficient volume of serum for AF-alb analysis, which includes 257 deceased cases, 612 survived hospital controls and 222 CPs. The percentage of missing samples was 7% for hospitalised (baseline), which is significantly lower than 18% for the CP group (p<0·001) and 27% for those deceased (p<0·001; online supplemental table 1).

**Table 1:** Demographic characteristics of the studied population

	Hospitalised CohortDeaths	Hospitalised CohortSurvivors	Community participants	Total
Sample size	257	612	222	1091
Gender, male (%)	139 (54%)	357 (58%)	122 (55%)	618 (57%)
Age (months)				
Mean±SD	11.5±5.6	11.5±6.0	12.5±6.0	11.7±5.9
Range	2.0–23.7	2.0–24.0	2.4–23.9	2.0–24.0
Weaning stage				
Exclusively breast fed, (%)	19 (7%)	58 (10%)	21 (9%)	97 (9%)
Partially breast fed, (%)	141 (55%)	336 (55%)	133 (60%)	610 (58%)
Fully weaned, (%)	97 (38%)	218 (36%)	68 (31%)	384 (35%)
Diet diversity score (range 0–12)				
Mean±SD	3.6±3.3	4.4±3.5	5.1±3.9	4.3±3.6
Nutritional strata**				
NW, (%)	30 (12%)	242 (40%)	NA	NA
MW, (%)	47 (18%)	157 (25%)		
SWK, (%)	180 (70%)	213 (35%)		
Socio-economic score (range 0–1)				
Mean±SD	0.5±0.3	0.5±0.3	0.5±0.2	0.5±0.3
Liver function marker				
ALT, mean±SD	40.1±67.7	38.6±94.8	76.3±261.0	46.6±141.8
ALK phosphate, mean±SD	244.9±143.9	234.7±142.0	237.1±152.7	237.5±144.2
Bilirubin, mean±SD	11.1±45.1	7.7±16.1	18.0±57.6	10.3±34.9

For some participants, only a sample at discharge was available. In subsequent analysis of AF-alb and mortality, only those with available admission samples were included.

* No nutrition strata in the community participant group. X2 test for prevalence difference between the two groups, p<0·001.

ALK, alkaline phosphatase; ALT, alanine transaminase; MW, moderately wasted; NW, not wasted; SWK, severely wasted or kwashiorkor.

For African participants, 10%, 52% and 38% of the participants were exclusively breastfed, partially breastfed and fully weaned, respectively, whereas the percentages were 6%, 63% and 31% in south Asian participants. X^2^ test showed significant correlation between weaning stages and which continent that the participants are from (p=0.001). The DDS was lower in African children than Asian children (mean±SD: 3·9±3·2 vs 5·3±4·0, p<0·001), as was the HED (0·4±0·2 vs 0·7±0·2, p<0·001). Detailed demographic data for each study site is summarised in online supplemental table 2.

### Serum AF-alb concentrations and determinants

**Figure 2:**
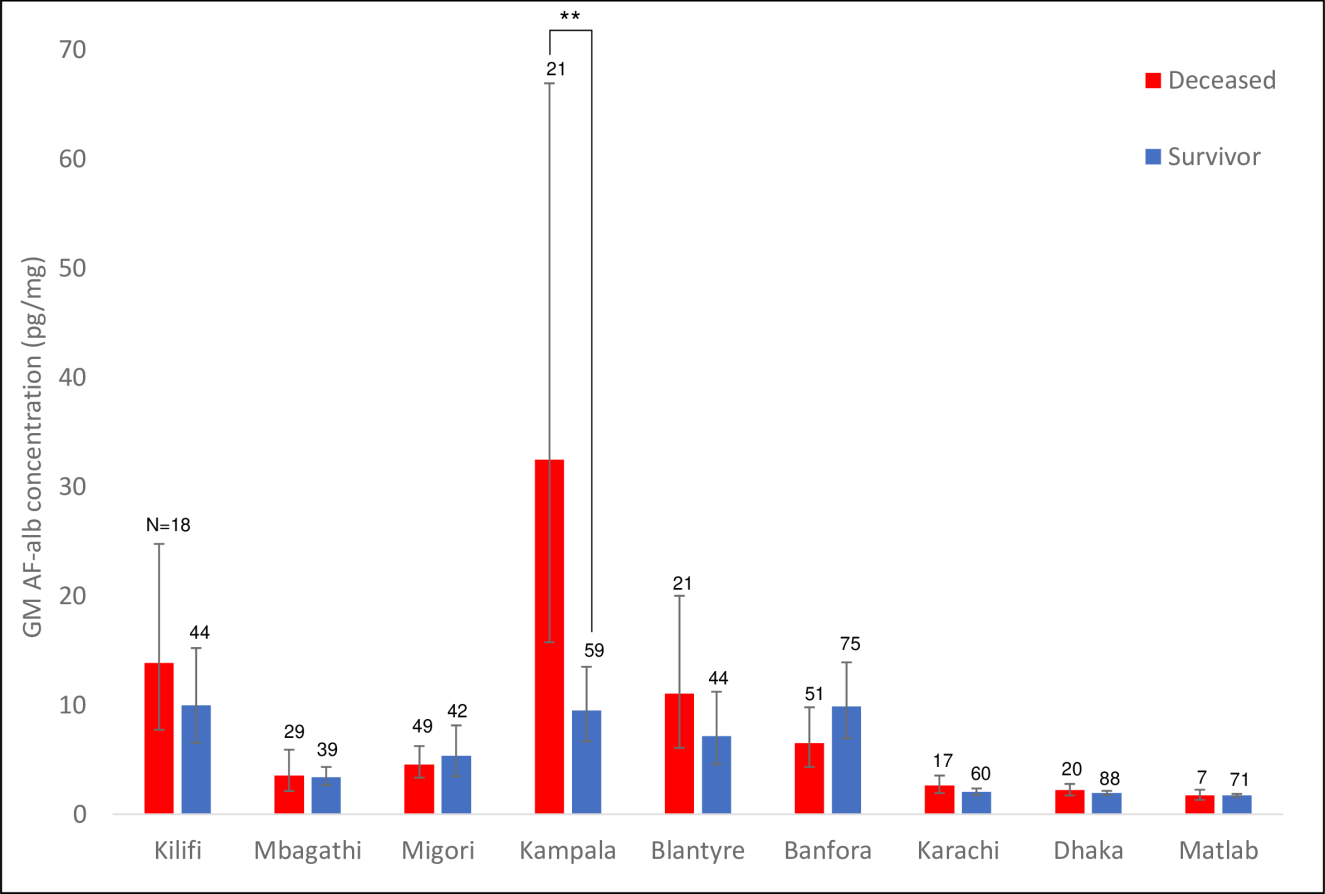
AF-alb concentrations between dead and alive groups by study site. *. p<0.05, **. p<0.01 ***. p<0.001, by independent t-test.

Serum AF-alb concentrations by the study site are shown in figure 2 with detailed data in online supplemental table 3.

Overall, 56%, 55% and 45% of the study participants tested positive for AF-alb in the admission, discharge and CP groups, respectively. The geometric mean (GM) and 95% CI were 4.7 (4·2 to 5·1), 3.7 (3·3 to 4·1) and 3·7 (3·1 to 4·3) pg/mg at admission, discharge and CP, respectively. The AF-alb levels were significantly higher at admission as compared with the CP group and those discharged (p=0·01). This difference between AF-alb at admission and discharge applied to both deaths and survivors.

Participants from Africa had consistently higher AF-alb-positive rates and GM levels than Asian children at admission, discharge and CP groups. The AF-alb-positive rate at admission was 71% vs 24% for African and Asian children, and the GM (95% CI) of AF-alb was 7·4 (6·5 to 8·5) vs 1·9 (1·8 to 2·1) pg/mg (p<0·01).

Among all the sites, children from Kampala showed the highest mean AF-alb levels at admission 13·1 (9·3 to 18·4) pg/mg and at discharge 10·7 (7·6 to 15·0) pg/mg (figure 2). The top four high aflatoxin exposure sites were consistently from Kampala, Uganda; Blantyre, Malawi; Banfora, Burkina Faso; and Kilifi, Kenya.

In total, 472 participants had paired AF-alb data available at both admission and discharge (hospital interval range: 0–46 days). AF-alb level was significantly reduced following hospitalisation (4.2 vs 3.5 pg/mg, p<0·02).

**Figure 3:**
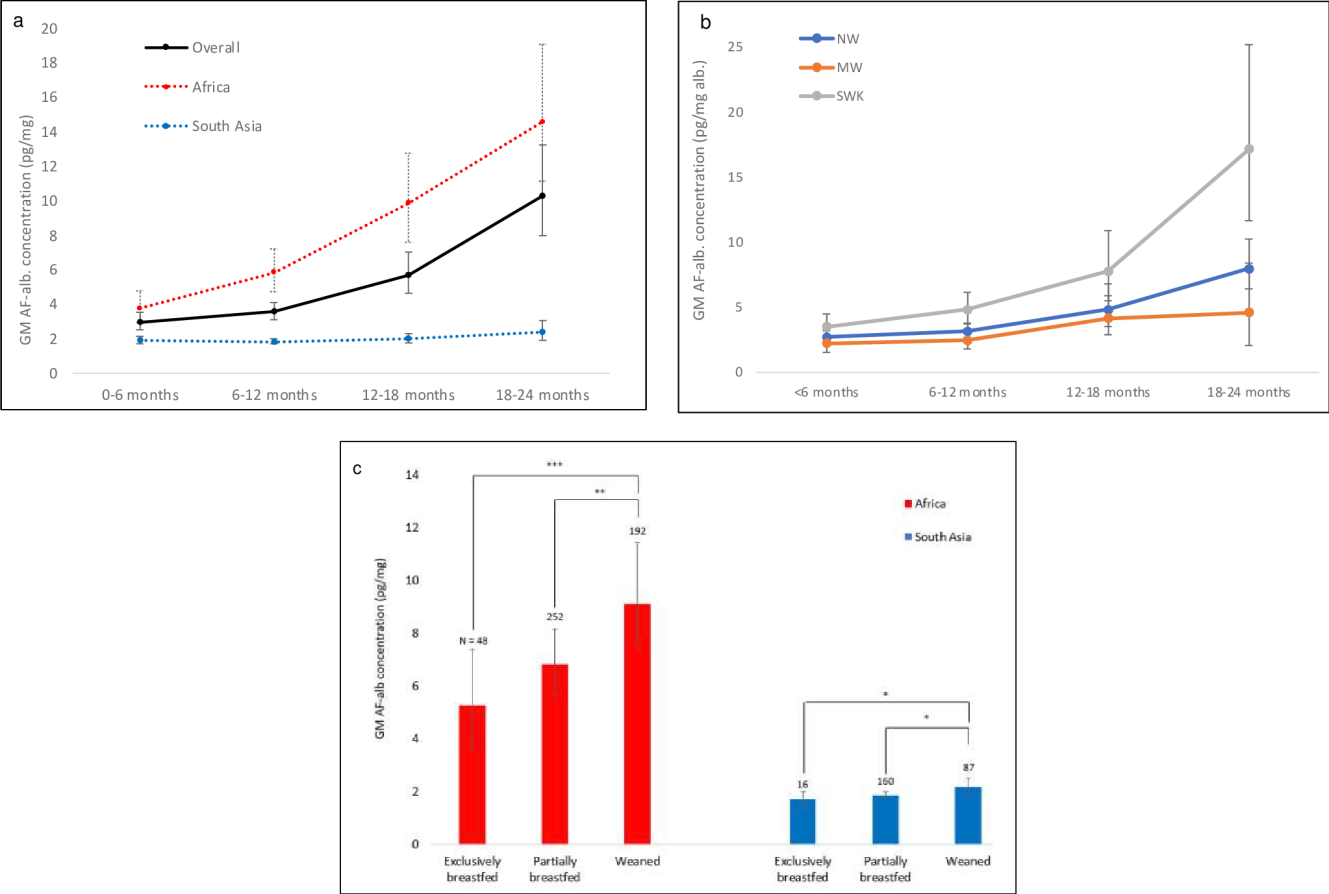
Key demographic factors that are significantly associated with participants’ serum AF-alb concen(a). Relationship between participants’ serum AF-alb concentrations and age groups by continent; (b). Relationship between participants’ serum AF-alb concentrations and age groups by nutritional strata (c). Relationship between participants’ serum AF-alb concentrations and weaning stages. * p<0.05 ** p<0.01 ***p<0.001 by independent sample t-test, weighted.

AF-alb concentrations increased with the child’s age (r=0·316, p<0·001); see figure 3a. No significant difference in AF-alb levels was found between gender groups. No association was found between AF-alb and food consumption frequency or DDS.

Among the three nutritional strata, AF-alb levels were 6·3 (5·4 to 7·4) pg/mg in the SWK group, significantly higher than the NW group at 4·1 (3·5 to 4·8, SWK v.s. NW: p<0·001) and the MW group 3·1 (2·6 to 3·6, SWK v.s. MW: p=0.043), respectively. The AF-alb levels by nutritional strata and age groups are shown in figure 3b.

For participants from Africa, the fully weaned group had significantly higher AF-alb concentrations as compared with the partially weaned and exclusively breastfed group (GM: 8·9 vs 6·9 pg/mg, p=0·003, and 8·9 vs 5·4 pg/mg, p<0·001; figure 3c). A similar, though less marked, trend was observed for participants from Asia (p<0·05).

A significant correlation was observed between AF-alb concentrations and HED scores (r = - 0·252, p<0·001), and participants in the higher HED quintiles had significantly lower levels of AF-alb compared with the participants in the lower HED quintiles, as shown in online supplemental figure 1.

There was a significant correlation between AF-alb levels and either alanine transferase (ALT; r=0.138, p<0.001) or bilirubin (r=0.1, p<0.001) using Spearman correlation analysis, but no significant correlation with alkaline phosphatase.

### AF-alb as a potential risk factor for mortality

There was no significant difference in gender, age, weaning stage, DDSs or HED between the deceased, the survival and CPs. Of the deceased group, 70% children were SWK, whereas there were just 35% SWK children in the survival group (p<0·001).

The mortality rate in the African children was 34%, significantly higher than that of the Asian children (19%, p<0·001).

The AF-alb level (pg/mg alb) was higher in the deceased (GM and 95% CI: 5·9 (4·9 to 7·1)) than in those who were hospitalised and survived (4·2 (3·8 to 4·7)] or in the CP [3·7 (3·1 to 4·3)). Data stratified by nutritional group (online supplemental table 4) showing the statistically significant difference in AF-alb levels between deaths and survivors is only seen for the NW group.

To examine the relationship between aflatoxin exposure and mortality, the odds of mortality of the participants were first compared between the AF-alb concentration quartiles, as shown in table 2. Overall, the AF-alb concentration quartile group was positively associated with mortality (p=0·003). However, after adjusting for site, age, gender, nutritional strata and HED and weighted for selection, the significant positive association between AF-alb and mortality disappeared.

**Table 2:** Logistic regression for the association between death and aflatoxin B_1_-albumin quartiles, not adjusted/adjusted for age, gender and household exposure domain score, site and nutrition strata, weighted for selection

AF-alb quartiles (pg/mg alb.)	Unadjusted model			Fully adjusted model		
	OR	95% CI	p value	OR	95% CI	p value
<1.5 lowest	1	Ref	Ref	1	Ref	Ref
1.5–3.4	1.05	0.39–2.84	0.928	0.93	0.32–2.68	0.894
3.4–9.4	1.43	0.87–2.35	0.164	1.04	0.59–1.81	0.905
> 9.4 highest	2.06	1.29–3.31	0.003**	1.24	0.68–2.27	0.481

*. *p* < 0·05, **. *p* < 0·01, ***. *p* < 0·001

The logistic regression model was then performed by separating the nutrition strata individually, adjusting for age, gender, HED and site, for which AF-alb level was associated with increased risk of mortality in NW children. As shown in table 3, the highest vs lowest AF-alb quartile group has an OR=4·84, p=0·014, in NW children only, but no such trend was found in the MW and SWK groups.

**Table 3:** Logistic regression for the association between death and aflatoxin B_1_-albumin adduct quartiles overall and by malnutrition group (adjusted by age, gender, household exposure domain score and site and weighted for selection)

AF-alb quartiles (pg/mg alb.)	NW (n=232)			MW ^a^ (n=173)			SWK ^a^ (n=350)			All (n=755)		
	OR	95% CI	p value	OR	95% CI	p value	OR	95% CI	p value	OR	95% CI	p value
<1.5 lowest	1	Ref	Ref	1	Ref	Ref	1	Ref	Ref	1	Ref	Ref
1.5–3.4	1.12	0.13–10.0	0.917	1.16	0.15–8.84	0.888	0.78	0.14–4.39	0.776	0.93	0.33–2.62	0.890
3.4–9.4	1.60	0.50–5.10	0.430	0.83	0.19–3.69	0.805	1.00	0.47–2.12	0.997	1.18	0.69–2.05	0.545
> 9.4 highest	4.84	1.38–17.0	0.014 **	0.18	0.01–2.47	0.198	0.97	0.44–2.17	0.972	1.84	1.02–3.30	0.043 *

*. *p* < 0·05, **. *p* < 0·01, ***. *p* < 0·001.

The association between death outcome and AF-alb by site is shown in figure 2. Children at the Kampala site had the highest aflatoxin exposure and had significant associations between AF-alb concentration and death outcome (OR 3·9, 95% CI 1·2 to 10·4).

## Discussion

Children admitted to hospital due to acute illness are at high risk of death, but factors that contribute to the mortality in these children are not completely understood. This study using the CHAIN cohort reveals that a higher level of AF-alb, a validated biomarker of aflatoxin exposure, was associated with high mortality among the NW children admitted to hospital, with the highest exposure quartile having 4·84-fold higher odds for mortality than those of the lowest exposure quartile (p=0·014). No such association was observed in children with moderate or severe wasting or when the model included all groups together. This likely reflects the dominant role of malnutrition as a risk factor for death, with aflatoxin exposure only being significant when moderate to severe wasting is absent.

These results only show an association between higher aflatoxin exposure and mortality in the non-wasted children, and it is too early to say whether or not this association is causal. It is known that aflatoxin exposure can cause liver toxicity, impair immune function^24^ and inhibit protein synthesis. Aflatoxin may interfere with intestinal integrity and hepatic metabolism and lead to malabsorption of nutrients and increased vulnerability to gut infections.^25^ Here, there was a correlation between AF-alb and two out of three markers of liver damage, ALT and bilirubin. All those factors could contribute to reduced nutrition metabolism, increased susceptibility to infection and decreased resilience against acute illness.^25^,^27^

Although the length of hospitalisation was not associated with aflatoxin levels in this analysis, there is a clear trend that aflatoxin biomarker levels were lowered at discharge as compared with admission, to a level that is similar to the CP group. This suggests that children in hospital may have reduced exposure to dietary aflatoxin from food received in hospital compared with their normal diet. Alternatively, the treatment received in the hospital may have improved liver function, interfered with the aflatoxin activation and/or increased detoxification.

Aflatoxin biomarker levels observed in this cohort were lower in children from Asian study sites compared with those from Africa, which is in keeping with previous studies.^28^ Maize, which is susceptible to aflatoxin contamination, is the main staple food in many African countries, while in Asia, rice and wheat are staples and these are less prone to aflatoxin contamination. The tropical temperature and agricultural practices (maize harvest and storage) are other factors that contribute to aflatoxin contamination level differences by region.^29^ The highest AF-alb levels were seen in Kampala, which is the only individual site to show a significant association between AF-alb and mortality. This may suggest that there is a threshold for the effect of aflatoxin on this endpoint, but more data is required to confirm this.

As has been previously reported, AF-alb was lower in children being breastfed than those fully weaned, and increased with age, as more family food is consumed.^24^ These highlight that the consumption of weaning food and family food results in a rapid increase in aflatoxin exposure risk in childhood. Although the AF-alb level was low for participants who were breastfed, the AF-alb level is still detectable. Maternal aflatoxin exposure can be transferred to the child.^30^ Therefore, it is also important to control the aflatoxin exposure of the mother. Although the food consumption frequency and DDS did not show any correlation with particular foods and aflatoxin exposure levels, it is likely the basic questions on food consumption frequency did not capture the information with sufficient sensitivity. It does not negate the important role of diet in aflatoxin exposure.

This study had several important strengths. Children were enrolled across a wide range of geographic and epidemiologic contexts and across a range of nutritional strata. Data and samples were systematically collected using standardised protocols across all sites, and loss to follow-up was minimal. However, there were also several limitations to this study. Some samples could not be used for biomarker determination due to insufficient serum being available. As a result, the missing samples were not evenly distributed across the death, survival and CP groups (online supplemental table 1). This, together with the fact that the study was focused on hospitalised patients, means the findings may not be able to be extrapolated to the general population.

## Conclusions

Among acutely ill hospitalised children in Africa and Asia, higher AF-alb biomarker levels were associated with mortality in non-wasted children but not in those with moderate to severe wasting. This risk was highest for children in sub-Saharan Africa, where exposure levels were highest. Exposure increased with age, reflecting when family food is introduced. While further study in the general population is merited, these findings highlight the continued importance of public health measures to reduce aflatoxin exposure. Identifying mechanisms linking aflatoxin to death may lead to the development of new interventions to reduce mortality among high-risk children in low- and middle-income settings.

## Supplementary material

10.1136/bmjgh-2024-017375 online supplemental file 1

## Data Availability

Data are available upon reasonable request.
