# Supplementary material for: Aflatoxin exposure and mortality in acutely ill children: results from the CHAIN network cohort
Source: BMJ Glob Health. 2025 Jul 17;10(7):e017375. doi: 10.1136/bmjgh-2024-017375 (PMC12273159; doi:10.1136/bmjgh-2024-017375)
Supplement: online supplemental file 1 [file bmjgh-10-7-s001.pdf]

Supplementary:

Table S1. Comparison of sample missing group and samples analysed for AF-alb group

	Missing samples	Samples with AF-alb data	Total
**Alive n (%)**	46 (7%)	612 (93%)	658
**Deceased n (%)**	93 (27%)	257 (73%)	350
**CP n (%)**	48 (18%)	222 (82%)	270

There is a significant association between if the sample is missing and the participant groups, $p < 0{\cdot}001$ by chi-square test, either between the alive and deceased group or between the alive and CP group.

Table S2 Demographic characteristic of the studied population by sites

Sites	Kilifi	Mbagathi	Migori	Kampala	Blantyre	Banfora	Karachi	Dhaka	Matlab	Total
**Sample size**	89	92	133	131	168	124	138	107	168	1091
**Gender, male (%)**	52 (58%)	54 (59%)	77 (58%)	77 (59%)	87 (52%)	68 (55%)	78 (57%)	63 (59%)	87 (52%)	618 (57%)
**Age**										
Mean ± SD	12·4 ± 6·2	11·1 ± 6·2	10·8 ± 6·2	11·9 ± 4·9	12·8 ± 6·1	11·0 ± 6·0	10·1 ± 5·4	11·4 ± 4·9	12·8 ± 6·1	11·7 ± 5·9
**Weaning stage**										
Exclusively breast fed, (%)	6 (7%)	12 (16%)	17 (13%)	7 (5%)	26 (18%)	9 (7%)	8 (6%)	8 (8%)	26 (18%)	101 (10%)
Partially breast fed, (%)	55 (66%)	49 (59%)	62 (47%)	51 (40%)	101 (70%)	56 (45%)	89 (65%)	89 (83%)	101 (70%)	610 (58%)
Fully weaned, (%)	23 (27%)	22 (27%)	54 (41%)	71 (55%)	18 (12%)	59 (48%)	41 (30%)	10 (9%)	18 (12%)	339 (32%)
**Diet diversity score [b]**										
Mean ± SD	2·3 ± 1·8	6·0 ± 4·3	4·3 ± 3·9	5·0 ± 2·9	2·4 ± 2·1	3·8 ± 3·5	4·0 ± 2·8	8·7 ± 4·1	2·4 ± 2·1	4·5 ± 3·6
Range	0-10	0-12	0-12	0-11	0-10	0-12	0-12	0-12	0-10	0-12
**Household exposures**										
Low, (%)	9 (13%)	32 (41%)	4 (4%)	32 (29%)	12 (12%)	12 (9%)	72 (77%)	77 (71%)	19 (24%)	267 (31%)
Medium, (%)	24 (33%)	37 (44%)	15 (15%)	50 (46%)	15 (18%)	58 (42%)	22 (23%)	20 (19%)	47 (60%)	286 (33%)
High, (%)	39 (54%)	13 (15%)	87 (81%)	28 (26%)	58 (70%)	68 (49%)	0 (0%)	11 (10%)	13 (17%)	316 (36%)

Table S3 AF-alb concentrations of the studied population by sites

	Sub-Saharan Africa						South Asia			Total[a]
**Country**	**Kenya**			**Uganda**	**Malawi**	**Burkina Faso**	**Pakistan**	**Bangladesh**		
**Site**	**Kilifi**	**Mbagathi**	**Migori**	**Kampala**	**Blantyre**	**Banfora**	**Karachi**	**Dhaka**	**Matlab**	
**Admission** ***										
Sample size	62	68	91	80	65	126	77	108	78	755
Positive, (%)	55 (89%)	36 (53%)	55 (60%)	69 (86%)	45 (69%)	89 (71%)	24 (31%)	28 (26%)	11 (14%)	412 (56%)
Range	1·5-507·7	1·5-736·0	1·5-237·4	1·5-732·2	1·5-254·7	1·5-1371·9	1·5-27·9	1·5-6·2	1·5-6·5	1·5-1371·9
Geometric mean (95%CI)	11·0 (7·8-15·5)	3·5 (2·7-4·5)	4·9 (3·8-6·3)	13·1 (9·3-18·4)	8·2 (5·7-11·8)	8·3 (6·4-10·9)	2·2 (1·9-2·5)	2·0 (1·8-2·2)	1·7 (1·6-1·9)	4·7 (4·2-5·1)
**Discharge** *										
Sample size	53	39	64	72	49	90	65	98	56	586
Positive, (%)	38 (72%)	17 (44%)	36 (56%)	57 (79%)	32 (65%)	57 (63%)	14 (22%)	12 (12%)	3 (5%)	266 (55%)
Range	1·5-601·6	1·5-14·3	1·5-75·1	1·5-309·2	1·5-153·6	1·5-849·1	1·5-8·3	1·5-5·8	1·5-4·2	1·5-849·1
Geometric mean (95%CI)	6·7 (4·5-9·8)	2·5 (2·0-3·1)	4·2 (3·2-5·6)	10·7 (7·6-15·0)	6·7 (4·5-9·9)	5·4 (4·2-7·0)	1·9 (1·7-2·1)	1·7 (1·6-1·8)	1·6 (1·5-1·7)	3·7 (3·3-4·1)
**Community participants** ***										
Sample size	17	14	26	21	26	30	30	30	28	222
Positive, (%)	17 (100%)	8 (57%)	12 (46%)	15 (71%)	23 (89%)	20 (67%)	1 (3%)	1 (3%)	2 (7·1%)	98 (45%)
Range	2·4-126·6	1·5-8·2	1·5-34·4	1·5-40·1	1·5-155·7	1·5-152·1	1·5-3·8	1·5-5·9	1·5-3·8	1·5-155·7
Geometric mean (95%CI)	15·3 (9·1-25·6)	3·0 (2·1-4·3)	3·1 (2·1-4·4)	5·2 (3·4-7·9)	12·9 (8·1-20·4)	6·7 (4·0-11·2)	1·6 (0·6-3·8)	1·6 (1·4-1·7)	1·6 (1·5-1·8)	3·7 (3·1-4·3)

*. *p*<0.05, **. *p*<0.01, ***. *p* < 0.001 when compared between sites using one-way ANOVA. Significant different AF-alb levels were found in participants from different sites at both admission, discharge and also among the community participants. Consistently Kampala, Blantyre, Banfora and Kilifi were on the top four highest exposure sites.

a*. Significant difference of AF-alb levels between admission and CP groups, t-test. *p* = 0.02; paired t test of AF-alb levels between admission and discharge t=5.33, *p* < 0.001.

Table S4 AF-alb concentrations of the studied population in different nutritional status group grouped by death outcomes.

	NW		MW		SWK	
	**Alive**	**Deceased**	**Alive**	**Deceased**	**Alive**	**Deceased**
**Sample number, n**	204	28	133	40	185	165
**AF-alb, GM (95 CI%, pg/mg)**	3.8 (3.2 – 4.5)	6.4 (3.9 – 10.6)	3.3 (2.7 – 4.0)	2.5 (2.0 – 3.2)	5.6 (4.5 – 7.0)	7.1 (5.6 – 9.0)

Note: Here, there are 522 survivor and 233 death samples, where the demographic described 612 survivor and 257 death samples. This is because there are 90 survivors, and 24 deceased participants only had their discharge blood sample available and are not included here.

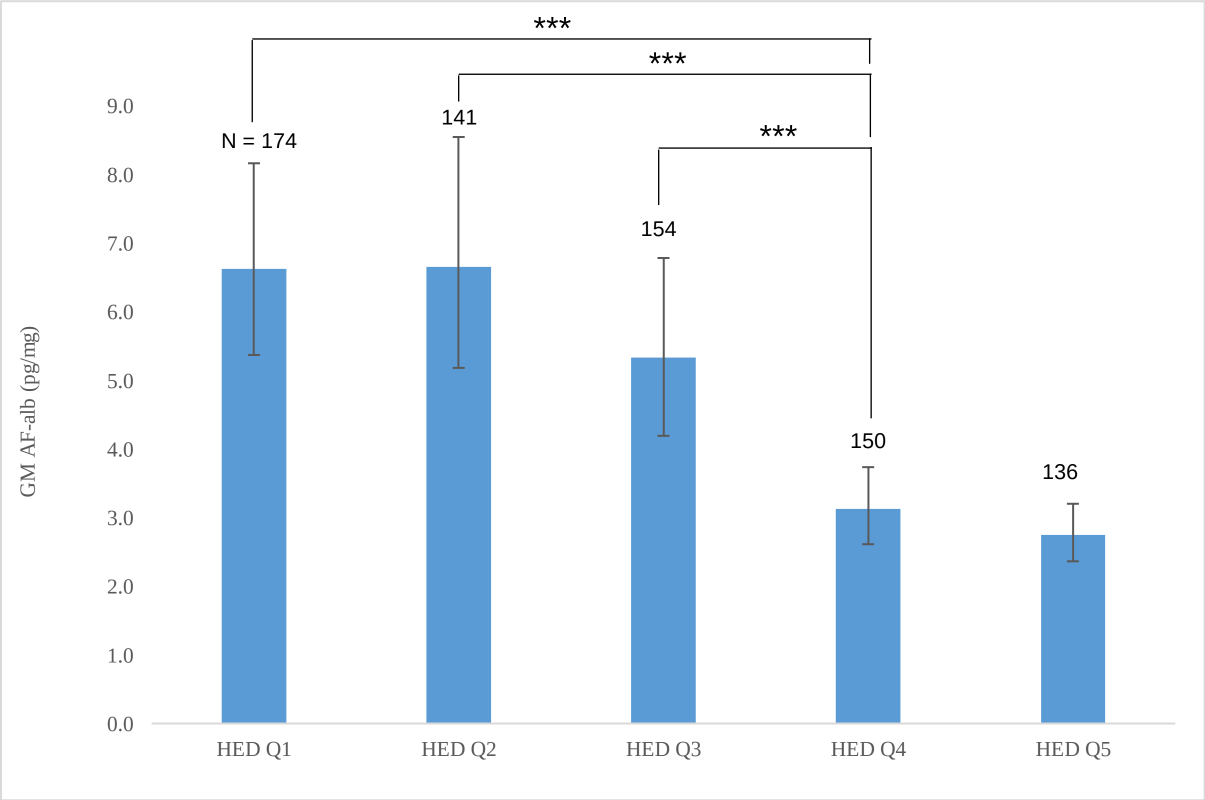

Figure S1 Distribution of AF-alb concentrations in different HED quintile groups. ***. $p<0.001$ by independent sample t-test.
